# *CORR* Insights^®^: Complications Are Not Increased With Acetabular Revision of Metal-on-metal Total Hip Arthroplasty

**DOI:** 10.1007/s11999-016-4870-8

**Published:** 2016-05-06

**Authors:** Stephen A. Jones

**Affiliations:** University Hospital of Wales, Heath Park, CF14 4XW Cardiff, UK

## Where Are We Now?

The past decade has been dominated by the rise and fall of metal-on-metal (MoM) as a bearing surface in THA. Unfortunately, the treatment of patients who have adverse local tissue reactions from MoM THA, specifically major soft-tissue damage, continues to present considerable challenges. The current study by Penrose and colleagues examined the Medicare database, detailing and comparing the outcome of revision for MoM and non-MoM THA. Their conclusions indicate similar high risks of dislocation, infection, and rerevision in both groups. This comparison to the more-traditional cohort of patients undergoing acetabular revision THA is certainly helpful in counseling affected patients, particularly with regard to infection risk, which has been a major issue in some series [3].

## Where Do We Need To Go?

Determining the etiology of these adverse events is the vital next step. Instability following primary and revision THA represents a leading cause of failure, which is once again demonstrated in this cohort. The specific challenges vary on a case-by-case basis, and there may well be vastly different reasons for high proportions of patient who experience dislocation in the two groups considered. Instability following THA is often multifactorial and we need to document and understand these risk factors and how they interact [1].

The MoM group itself can in fact present a diverse group, in which some of the patients may have been identified through a recall program at an early stage in the natural history of adverse local tissue reactions. One would anticipate that patients with this history might be at less perioperative risk that patients presenting later with massive abductor muscle destruction. Bearing failure in MoM is also “clouded” by the influence of taper failure in this cohort, and our understanding of these complex mechanical as biological mechanisms continue to evolve [2].

## How Do We Get There?

Many centers that embraced MoM did so in large numbers, resulting in a robust recall and monitoring process for affected patients. In order to reduce complication rates and improve outcomes on-going surveillance and reporting from these groups will be necessary. Investigation into the natural history of bearing failure in MoM implants needs to continue to inform our clinical decision making for this patient population. The large data sets provided by national joint registries were pivotal in the in the initial identification of high early failure rates with some MoM implants. This of course led to the recall of certain MoM implant designs. Moving forward registry data will be particularly helpful to detail the outcome of implant designs that are utilized to prevent and treat instability, namely dual mobility and Constrained Acetabular Liners (CALs).

Regarding dislocation, we need to focus our efforts on understanding how risk factors interact: Is it additive or multiplicative? For those risk factors that cannot be modified, a proven method such as risk stratification could aid implant selection and help prevent dislocation. The current indications for the use of dual mobility bearings and CALs remains unclear. We need to better understand the group of patients that would most benefit from these implants. It may be that these implants are to be considered as salvage devices, but what we need to accurately define what constitutes a “salvage patient” particularly regarding the spectrum of soft-tissue loss around the hip. In many cases, these deficiencies can range from localized damage to the posterior capsule and short rotators to massive abductor loss.

## References

[1] Jones SA. The prevention and treatment of dislocation following total hip arthroplasty: efforts to date and future strategies. *Hip Int*. 25;4:2015. 10.5301/hipint.5000273 26044529

[2] Plummer DR, Berger RA, Paprosky WG, Sporer SM, Jacobs JJ, Della Valle CJ (2016). Diagnosis and management of adverse local tissue reactions secondary to corrosion at the head-neck junction in patients with metal on polyethylene bearings. J Arthroplasty..

[3] Wyles CC, Van Demark RE, 3rd, Sierra RJ, Trousdale RT. High rate of infection after aseptic revision of failed metal-on-metal total hip arthroplasty. *Clin Orthop Relat Res*. 2014;472:509–516. 10.1007/s11999-013-3157-6 PMC3890189 23846604

